# Functional and structural analysis of missense variants in the human *PDCD1* Gene

**DOI:** 10.4102/jphia.v16i4.1348

**Published:** 2025-06-20

**Authors:** Hanâ Baba, Meryem Bouqdayr, Anass Abbad, Asmae Saih, Benson R. Kidenya, Mohamed A. Sesay, Simpson Addo, Lahcen Wakrim, Anass Kettani

**Affiliations:** 1 Biotechnology R&D Unit, Institut Pasteur du Maroc, Casablanca, Morocco; 2 Laboratory of Biology and Health, Faculty of Sciences Ben M’Sik, Hassan II University, Casablanca, Morocco; 3 Medical Virology and BSL-3+ Laboratory, Institut Pasteur du Maroc, Casablanca, Morocco; 4 Virology Unit, Immuno-virology Laboratory, Institut Pasteur du Maroc, Casablanca, Morocco; 5 Department of Biochemistry and Molecular Biology, Catholic University of Health and Allied Sciences, Mwanza, United Republic of Tanzania; 6 National Hepatitis Control Program, Ministry of Health, Freetown, Sierra Leone; 7 Government Hospital Viral Hemorrhagic Fever Research Site, Kenema, Sierra Leone; 8 Research, Statistics, and Information Management Directorate, Ministry of Health, Accra, Ghana

**Keywords:** programmed death-1 (PD-1), non-synonymous single nucleotide polymorphisms (nsSNPs), *in-silico* analysis, molecular dynamics (MD) simulations, bioinformatics

## Abstract

**Background:**

Programmed death-1 (PD-1) is an immune checkpoint receptor that regulates T-cell function by modulating and terminating immune responses.

**Aim:**

This study investigates the functional and structural impact of missense single nucleotide polymorphisms in the human *Programmed Cell Death 1* (*PDCD1*) gene.

**Setting:**

The data related to *PDCD1* gene single nucleotide polymorphisms [SNPs] were collected from dbSNP.

**Methods:**

PredictSNP1.0, integrating eight tools (sorting intolerant from tolerant [SIFT], PolyPhen-1/2, multivariate analysis of protein polymorphism [MAPP], predictor of human deleterious [PhD] SNP, screening for non-acceptable polymorphisms [SNAP], PANTHER, nsSNPAnalyzer), was used for variant predictions. Conservation was assessed with ConSurf, stability with MUPro and I-Mutant 2.0 and pathogenicity with MutPred2. Molecular dynamics (MD) simulations analysed native and mutant PD-1 variants over 100 nanosecond (ns), assessing root-mean-square deviation (RMSD), root-mean-square fluctuation (RMSF), radius of gyration (*R*_g_), solvent-accessible surface area (SASA) and hydrogen bonding.

**Results:**

D117V and W286G were identified as the most deleterious variants. However, W286G was located in an unfavourable structural region, rendering its model unreliable and excluding it from further analysis. Molecular dynamic simulations on the native and D117V models showed no significant differences in RMSD, RMSF, *R*_g_, SASA or hydrogen bonding, suggesting D117V (rs772130993) has minimal impact on PD-1 stability or flexibility.

**Conclusion:**

Bioinformatics tools predicted the D117V variant as deleterious, but molecular dynamics simulations suggest it may have limited functional impact.

**Contribution:**

These findings underscore the importance of integrating computational predictions with experimental validation to guide therapeutic exploration of genetic variants.

## Introduction

The *Programmed Cell Death 1* (*PDCD1*) gene, located on chromosome 2q37.3, encodes the Programmed Death 1 (PD-1) protein, a critical immune checkpoint receptor. Programmed death-1 is expressed on the surface of T-cells and plays a pivotal role in down-regulating immune responses and promoting self-tolerance by modulating T-cell activity.^1,2^ This occurs through its interaction with its ligands, PD-L1 and PD-L2, which are frequently overexpressed in various cancer cells, enabling tumours to evade immune surveillance.^3,4^ Therefore, inhibition of the PD-1/PD-L1 pathway has become a crucial strategy in cancer immunotherapy, with several PD-1 and PD-L1 inhibitors already approved for clinical use.^4,5^ This pathway is also essential in preventing autoimmunity, but its dysregulation is associated with several autoimmune diseases, such as systemic lupus erythematosus and rheumatoid arthritis. Moreover, T-cell exhaustion by the PD-1/PD-L1 pathway has a role to play in maintaining peripheral tolerance, but also in regulating antimicrobial defence. Therefore, the PD-1/PD-L1 pathway could limit the damage to the body caused by the exacerbated activation of the immune system during viral infections.^6^ Conversely, the suppression of antimicrobial defences facilitates the establishment of persistent infections. Notably, several viruses responsible for chronic infections, such as human immunodeficiency virus (HIV)-1, appear to exploit the PD-1/PD-L1 pathway to evade immune detection and establish long-term persistence.^2^

Given the crucial role of PD-1 protein in immune regulation, understanding the impact of genetic variations in the *PDCD1* gene is of significant interest. Therefore, investigating the most prevalent form of genetic variation in the human genome – non-synonymous single nucleotide polymorphisms (nsSNPs) – is of critical scientific importance, as these variants induce amino acid substitutions that may profoundly affect protein structure, stability and hence function. These variations can disrupt normal cellular processes and are often implicated in the pathogenesis of various diseases.^7,8^ Consequently, nsSNPs in *PDCD1* gene may alter the PD-1 protein’s structure, stability or interaction with its ligands, potentially leading to altered immune responses. These effects can be efficiently assessed using *in silico* approaches, which offer a cost-effective and powerful means of predicting the functional consequences of nsSNPs. By combining diverse data sources, including evolutionary conservation, amino acid physicochemical properties and structural information, computational tools offer valuable insights into the effects of genetic variations.^9^ Identifying and characterising deleterious nsSNPs not only enhances our understanding of the molecular mechanisms underlying various diseases but also informs the development of targeted therapeutic strategies. In the context of immunotherapy, understanding the functional consequences of PD-1 variants is particularly crucial for advancing precision medicine. Programmed death-1 inhibitors have revolutionised cancer treatment, particularly in melanoma, lung cancer and other malignancies, by enhancing anti-tumour immune responses.^10,11^ However, genetic variations in *PDCD1* gene may influence individual responses to these therapies, affecting efficacy and the risk of immune-related adverse events.^12^ Furthermore, in infectious diseases, such as HIV, where PD-1 overexpression contributes to T-cell exhaustion, genetic predispositions could inform personalised therapeutic strategies.^13^ Identifying functionally significant PD-1 variants could help stratify patients for immunotherapy, optimise treatment outcomes and reduce the burden of immune-related complications. This study contributes to a growing body of research supporting the integration of genetic screening in immunotherapeutic decision-making.^11^ Therefore, in this study, we conducted an *in silico* analysis to identify and characterise deleterious nsSNPs in the human *PDCD1* gene. By leveraging multiple computational prediction tools, we evaluated the potential functional, structural and dynamic impacts of these variants, as well as their pathogenicity on the PD-1 protein. Our findings aim to deepen the understanding of *PDCD1* genetic variants and implications thereof for human health, offering valuable insights that could contribute to the development of personalised therapeutic strategies.

## Research methods and design

### Prediction of the deleterious effect of non-synonymous single nucleotide polymorphisms of the *Programmed Cell Death 1* gene

#### Data collection

The amino acid sequence of the PD-1 protein in Fasta format was retrieved from the Uniprot database (https://www.uniprot.org) (UniprotKB ID Q15116). The data related to *PDCD1* gene SNPs were collected from dbSNP (http://www.ncbi.nlm.nih.gov/snp/). A table of variants was realised where we filtered common variants and then classified them in order of priority to identify those which can potentially affect the protein function.

#### Selection of deleterious non-synonymous single nucleotide polymorphisms

To predict the deleterious effect of nsSNPs of the human *PDCD1* gene on protein function, different bioinformatics tools were used. We employed the PredictSNP1.0 (http://loschmidt.chemi.muni.cz/predictsnp1/) for prediction consensus classifier of disease-related mutations. PredictSNP1.0 was chosen for this study because of its comprehensive ability to predict the pathogenicity of single nucleotide polymorphisms (SNPs) by combining eight most powerful predictive algorithms: sorting intolerant from tolerant (SIFT), PolyPhen-1, PolyPhen-2, multivariate analysis of protein polymorphism (MAPP), predictor of human deleterious (PhD)-SNP, screening for non-acceptable polymorphisms (SNAP), PANTHER and nsSNPAnalyzer.^14^ This platform offers a reliable consensus prediction and integrates results from various tools, allowing for more accurate and consistent identification of potentially harmful nsSNPs. Compared to other computational tools, PredictSNP1.0 is particularly effective in assessing the functional impact of genetic variants, which makes it a suitable choice for this study. With this approach, we generated scores from each computational tool, accompanied by consensus predictions expressed as percentages, derived from observed accuracy values to facilitate straightforward comparisons.^14^

#### Evolutionary conservation analysis of programmed death-1

Conservation prediction of the PD-1 protein sequence was analysed with ConSurf (http://consurf.tau.ac.il/), a web server used for calculating the evolutionary conservation of nucleic acid or amino acid positions through phylogenetic relationships between homologous sequences. The algorithm of this tool predicts the important functional regions of a protein by estimating the conservation degree of amino acids on a scale of 1 to 9 based on multiple sequence alignment (MSA). Therefore, 9 represents the most highly conserved residue and 1 represents the least conserved region. This tool also analyses conservation at the nucleotide level. Polymorphisms located in conserved regions are likely to be more deleterious compared to those located in variable regions, as they tend to destabilise protein structure and function.^15^

#### Effect on the stability of programmed death-1 protein

The stability of the PD-1 protein was evaluated using the bioinformatics tools MUPro (http://mupro.proteomics.ics.uci.edu/) and I.Mutant 2.0 (http://gpcr2.biocomp.unibo.it/cgi/predictors/I-Mutant3.0/I-Mutant3.0.cgi), which have the advantage of not requiring tertiary structures for their calculations. MUPro predicts the effect of single amino acid changes on protein stability, providing a confidence score ranging from −1 to +1, where a score < 0 indicates decreased stability and a score > 0 indicates increased stability.^16^ Similarly, I.Mutant 2.0 assesses the impact of mutations on protein stability by calculating the Gibbs free energy change (DDG) resulting from amino acid substitutions, with DDG values also ranging from −1 to +1. A positive DDG (> 0) signifies increased stability, while a negative DDG (< 0) indicates decreased stability.^17^ Together, these tools offer quantitative insights into the effects of specific mutations on PD-1 protein stability, providing valuable data for understanding the functional consequences of genetic variations.^18^

#### Pathogenicity prediction of mutations

Pathogenicity predictions were conducted using MutPred2 (Mutation Prediction2) (http://mutpred.mutdb.or/help.html), a machine learning-based tool that integrates genetic and molecular data to estimate the pathogenic potential of amino acid substitutions. MutPred2 predicts the gain or loss of structural and functional properties associated with a mutation. This tool requires only the protein sequence and the specific amino acid substitution as input. It generates scores ranging from 0 (benign) to 1 (pathogenic), along with a *p*-value that indicates the statistical confidence of the prediction. Outputs with a *p*-value < 0.05 are considered significant, while those with a *p*-value < 0.01 are deemed highly significant, reflecting greater confidence in the pathogenicity prediction. MutPred2 provides insights into the functional and structural consequences of mutations, offering valuable information for understanding their potential impact on protein behaviour.^19^

#### Structural analysis of the programmed death-1 protein

Proteins are inherently dynamic entities, characterised by the flexibility of their constituent amino acids and their diffusion within the surrounding environment. Likewise, ligands and solvents exhibit their own dynamic behaviours. Molecular dynamics (MD) simulations provide a powerful approach to model and analyse the time-dependent behaviour of these molecular systems.^20^

#### Preparation of the 3D structure of programmed death-1

Since the complete 3D structure of the PD-1 protein was not available in the Protein Data Bank (PDB), we resorted to homology modelling, based on the assumption that molecules with homologous sequences share similar structures. In this study, we used Iterative Threading ASSEmbly Refinement (I-TASSER, https://zhanggroup.org/I-TASSER/) to generate the complete 3D structure of PD-1. I-TASSER selects the model with the highest significance of thread alignments and the quality of the predicted structure is assessed using a confidence score (C-score), which ranges from –5 to +2, with higher values indicating greater confidence in the prediction. The C-score is correlated with the model’s template modelling score (TM-score) and the root-mean-square deviation (RMSD). The TM-score, a scale recently introduced to address local error issues with RMSD, ranges from 0 to 1, with a value of 1 indicating a higher structural similarity to improve the accuracy of the generated model and bring it closer to experimental levels. For further computational studies, we refined the PD-1 structure using the ModRefiner algorithm (https://zhanglab.ccmb.med.umich.edu/ModRefiner),^21^ which refines the backbone topology from the C-alpha traces and adds side chains based on a physics- and knowledge-based force field.^22^ For constructing the mutated models, mutagenesis was performed using PyMOL (http://www.pymol.org)^23^ and the 3D structures of the native and mutated proteins were evaluated using the Structural Analysis and Verification Server (http://nihserver.mbi.ucla.edu/SAVES/), employing Verify3D and PROCHECK programmes.^24^

### Molecular dynamics analysis

In this study, GROMACS 5.1.3 was selected for MDs simulations because of its efficiency, reliability and widespread use in molecular modelling.^25,26^ A 100-nanosecond (ns) MD simulation was conducted on the selected native and mutated 3D structures. The CHARMM (Chemistry at Harvard Macromolecular Mechanics) force field was employed for its versatility and proven accuracy in modelling macromolecular systems using empirical energy functions. Widely recognised as a leading tool for studying the MDs of biological molecules, particularly proteins, CHARMM provided a robust framework for this analysis.^27^

The homology-generated model served as the starting point for constructing the protein topology. The protein was placed in an aqueous environment within a cubic water box, with a 1 nm buffer surrounding the structure, to simulate physiological conditions.^28^ The system was neutralised by adding sufficient Na^+^ and Cl^-^ ions. Energy minimisation was conducted using the steepest descent algorithm for up to 50 000 steps to relax the solvated system and achieve a stable conformation.

After energy minimisation, the system was equilibrated in two stages. Firstly, an NVT (constant number of particles, volume and temperature) ensemble was run using the Berendsen thermostat to heat the system to 300 K and achieve a density of 1000 kg/m^3^. Secondly, an NPT (constant number of particles, pressure and temperature) ensemble was carried out at 1 bar and 300 K to ensure system stabilisation. Each equilibration phase lasted 500 ps.^29^

Following equilibration, 100 ns MD simulations were performed for both native and mutant models (wild-type [WT] and D117V). The resulting trajectory files were analysed using various GROMACS utilities, including g_rms, g_rmsf, g_hbond, g_mdmat, g_gyrate and g_sasa, to compute parameters RMSD, root-mean-square fluctuation (RMSF), hydrogen bonds (H-bonds), interaction residues, radius of gyration (*R*_g_) and solvent-accessible surface area (SASA), respectively.

The data generated from these analyses were visualised using X Window System Multi-platform GRaphing tool (XMGRACE) for plotting^30^ and Yet Another Scientific Artificial Reality Application (YASARA) View for molecular interaction representations.^31^ These analyses provided insights into the structural and dynamic behaviour of both native and D117V mutant PD-1 protein model under simulated physiological conditions.

## Results

### Distribution of *Programmed Cell Death 1* gene single nucleotide polymorphisms dataset

The complete dataset, retrieved from the dbSNP database, comprised 4478 SNPs, including 2287 intronic, 815 downstream, 452 located in the 3′UTR region, 214 nsSNPs, 22 in the 5′UTR region and 688 of other types. These nsSNPs were selected to investigate their potential deleterious effects on the function of the PD-1 protein. By focusing on the nsSNPs, we aimed to assess their possible impact on PD-1 functionality.

### Prediction of deleterious non-synonymous single nucleotide polymorphisms in *Programmed Cell Death 1* gene

Following the submission of 214 nsSNPs to PredictSNP1.0, a comprehensive in silico analysis was conducted using all available integrated prediction tools to evaluate their potential deleterious effects on pathogenicity.

**FIGURE 1:**
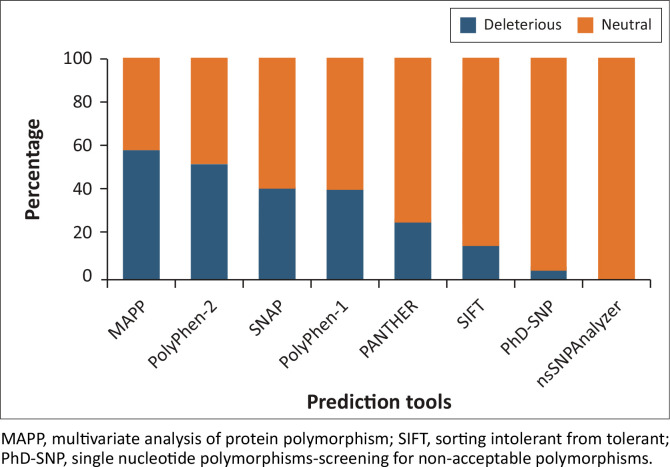
A graphical representation showing the distribution of 214 non-synonymous single nucleotide polymorphisms predicted as deleterious (in orange) and neutral (in blue) by PredictSNP1.0 server.

The results from MAPP predicted 135 out of 214 nsSNPs (63.1%) to be deleterious, followed by PolyPhen-2 with 110 deleterious nsSNPs (51.4%), SNAP with 91 nsSNPs (42.5%), PolyPhen-1 with 85 nsSNPs (39.7%), PredictSNP with 84 nsSNPs (39.2%), PANTHER with 55 nsSNPs (25.7%), SIFT with 38 nsSNPs (17.8%), PhD-SNP with 12 nsSNPs (5.6%) and nsSNPAnalyser with 0 deleterious predictions (0%) as shown in Figure 1.

Among these, two nsSNPs, D117V (rs77213099) and W286G (rs77510030), were consistently predicted to be deleterious by six out of the seven prediction tools used. These variants are highlighted in Table 1 as the most likely to impact the function of the PD-1 protein.

**TABLE 1:** Prediction scores of deleterious single nucleotide polymorphisms in the *Programmed Cell Death 1* gene by PredictSNP1.0.

nsSNP	Polymorphism	PredictSNP	SIFT	PolyPhen2	PolyPhen1	MAPP	SNAP	PANTHER	PhD-SNP
D117V	rs 77213099	0.82	0.79	0.81	0.74	0.77	0.85	0.61	0.85
W286G	rs 77510030	0.58	0.79	0.67	0.74	0.82	0.80	0.69	0.81

**SNP, single nucleotide polymorphism; nsSNP, non-synonymous single nucleotide polymorphisms;** SIFT, **sorting intolerant from tolerant; MAPP, multivariate analysis of protein polymorphism; SNAP, screening for non-acceptable polymorphisms; PhD, predictor of human deleterious.**

### Phylogenetic conservation

**FIGURE 2:**
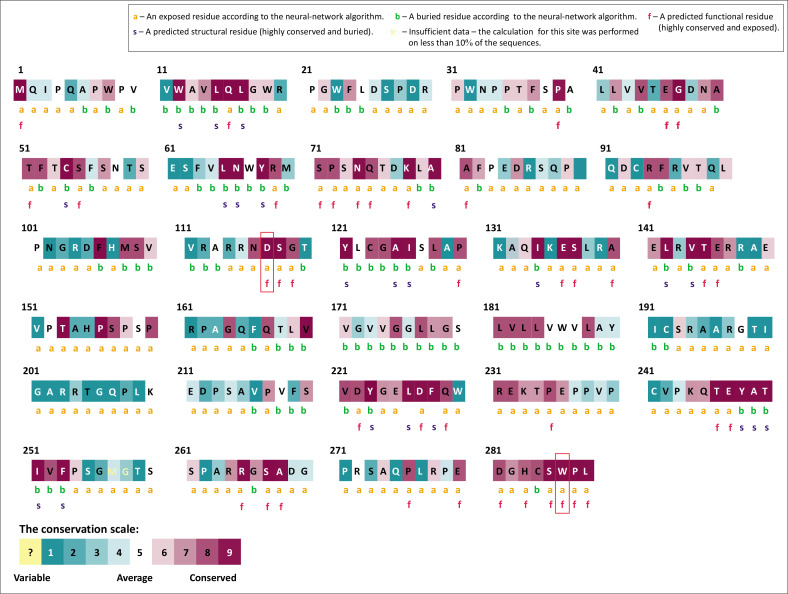
Results of programmed death-1 protein conservation analysis *via* ConSurf.

Using the ConSurf web server, we calculated the evolutionary conservation score of amino acid residues of the PD-1 protein to explore the possible effects of the two most deleterious nsSNPs. Our results showed that the two selected deleterious nsSNPs (D117V and W286G) were in highly conserved regions, exposed, functional and with a conservation degree of 9 (Figure 2).

### Impact of predicted deleterious mutations on programmed death-1 stability

The protein stability analysis of selected SNPs in PD-1 (D117V and W286G) using I-Mutant 2.0 and MUPro reveals a decrease in stability for both the mutation types. For D117V, both tools predict a decrease in stability with I-Mutant showing a negative DDG of −0.40 and MUPro indicating a similar effect across all models (support vector machine [SVM] and artificial neural network [NN]). Similarly, W286G shows a marked decrease in stability, with I-Mutant and MUPro (DDG −2.03) consistently suggesting a destabilising effect. The results are supported by reliability indices (RI) and predictive models (SVM and NN) (Table 2).

**TABLE 2:** Protein stability analysis results of selected single nucleotide polymorphisms of programmed death-1 protein using I-Mutant 2.0 and MUPro.

Position	I-Mutant 2.0			MUPro			
	DDG	RI	Effect on stability	DDG	SVM	NN	Effect on stability
D117V	−0.40	5	Decrease	−0.73	−0.28	−0.62	Decrease
W286G	−2.03	9	Decrease	−2.03	−1.0	−0.88	Decrease

DDG, free energy variation; RI, reliability index; SVM, support vector machine; NN, artificial neural network.

### Prediction of mutation-induced pathogenicity

MutPred2 prediction results showed that the pre-selected D117V and W286G nsSNPs scored 0.805 and 0.572, respectively, suggesting that they could be pathogenic rather than benign variants. Among these, the D117V polymorphism was the only one predicted to cause structural and functional alterations in the PD-1 protein. These alterations include a gain of adenosine diphosphate (ADP)-ribosylation at R112, an altered disordered interface, a modified transmembrane protein and a gain of N-glycosylation at N116, potentially affecting the protein’s stability and function (Table 3).

**TABLE 3:** Effects of nsSNP D117V on structural and functional properties of programmed death-1 protein resulting from MutPred2.

Molecular mechanisms	Probability of the polymorphism	*p*
Gain of ADP-ribosylation at R112	0.22	0.02
Altered disordered interface	0.17	0.04
Modified transmembrane protein	0.15	0.02
Gain of N-glycosylation at N116	0.07	0.02

ADP, adenosine diphosphate.

### Preparation and validation of programmed death-1 models

Although different polymorphisms in the PD-1 protein are reported, the native 3D structure with the complete sequence of 288 amino acids is not yet available on the PDB database. Besides, not all mutant protein structures are analysed and deposited in databases. Therefore, we were interested in our study in modelling the 3D structure of the native PD-1 protein and the mutant proteins in order to be able to compare them structurally and functionally. For this, the I-TASSER web server is deployed using as a homology model the primary sequence of PD-1 obtained from the National Center for Biotechnology Information (NCBI) database.

I-TASSER produced five models, from which we selected only the model with the highest minimum C-score, which suggests higher confidence of the predicted model. The TM-score of our chosen predicted secondary structure highlights the correct topology of the model. The C-score reflects the high quality of our predicted secondary structure and directly correlates with the TM-score and RMSD (Table 4).

**TABLE 4:** I-TASSER results representing the C-score, template modelling-score and root-mean-square deviation of the selected secondary structure.

Model number	C-score	TM-score	RMSD	Number of lures	Cluster density
Model 1	−3.23	0.35 ± 0.12	13.9 ± 3.9	1564	0.0215
Model 2	−3.42	-	-	1223	0.0178
Model 3	−3.51	-	-	991	0.0163
Model 4	−3.97	-	-	707	0.0103
Model 5	−3.87	-	-	750	0.0114

TM, template modelling; RMSD, root-mean-square deviation; TM-score, template modelling score.

**FIGURE 3:**
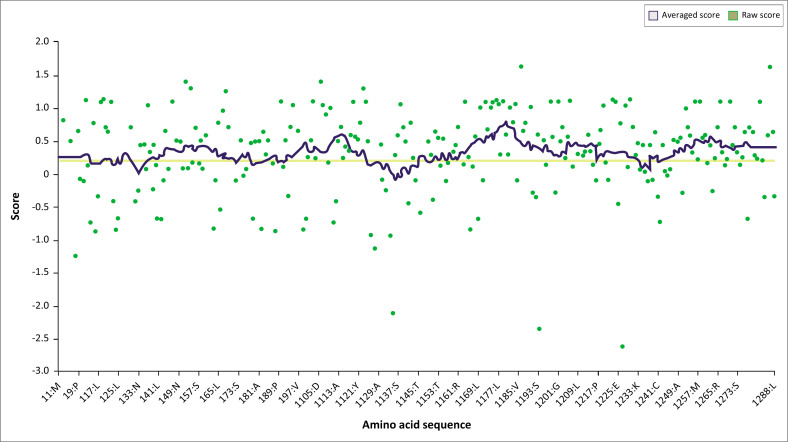
Programmed death-1 native 3D structure evaluation graph performed by Verify3D.

The generated 3D structure was refined using the ModRefiner server with an RMSD value of 1.071 and a TM-score of 0.9729 to the initial model. After refining our native structure, *via* PyMol, we constructed two mutated structures of the PD-1 protein with the two selected nsSNPs (D117V and W286G). All these 3D structures (native and mutated) were validated using the Verify3D and PROCHECK tools. Verify3D showed that 82.64% of the amino acids have a score ≥ 0.2 by comparing their 3D profile to the 1D, whether for the native or mutated structure (Figure 3). This means that according to Verify3D our structure is validated.

**FIGURE 4:**
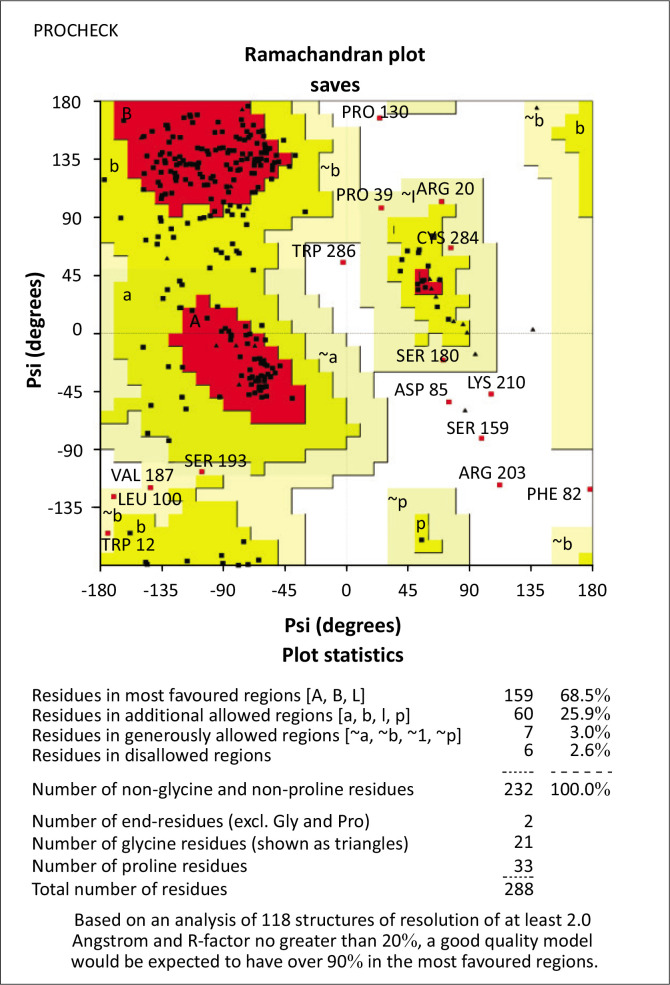
Ramachandran plot of PROCHECK validation of the native 3D structure of programmed death-1.

For our native structure, Ramachandran plot analysis by PROCHECK showed 159 (68.5%) residues in the favoured region, 62 (25.9%) residues in the additional allowed region, 7 (3%) residues in the generously authorised region and 6 (2.6%) residues in the unauthorised region. Plotting our predicted structure provided a view of the set of excluded regions that show the position of amino acids that are not allowed. This is the case for the 6 residues (Proline130, Tryptophan286, Lysine210, Aspartate85, Serine159 and Arginine203) which were found at very long distances from the predicted model pocket, suggesting that these amino acids are structurally and functionally irrelevant (Figure 4).

Concerning the validation by PROCHECK of the mutated structures (D117V and W286G), the estimation of the stereochemical quality showed that the D117V structure is reliable and valid for further MDs calculations (Figure 4).

Alternatively, the W286G structure is proposed to be irrelevant because of the fact that residue 286 before and after mutagenesis is located in an unauthorised or unfavourable region (Figure 4). These results allowed us to exclude the possibility of exploiting the mutated W286G model in the continuation of our study.

### Molecular dynamics simulations

#### Root-means-square deviation

**FIGURE 5:**
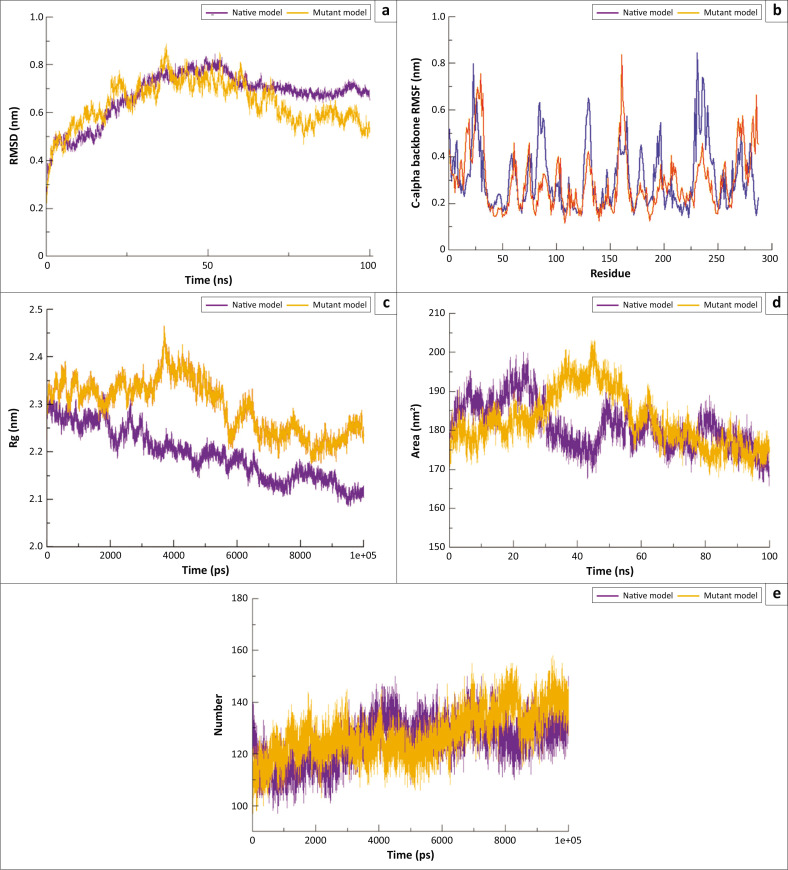
Molecular dynamics (MD) simulation analysis with: (a) root-mean-square deviation, (b) root-mean-square fluctuation, (c) *R*_g_, (d) solvent-accessible surface area and (e) H-bond values across Cα backbone (in Å) of native (WT) and mutant (D117V) models calculated after 100 ns of MD trajectories.

The conformational stability of the native and mutated PD-1 protein models (WT vs. D117V) during the MD simulation was evaluated using RMSD analysis. The native model exhibited an average RMSD value of 0.670 nm ± 0.103 nm, which was closely comparable to the D117V mutated model, with an RMSD of 0.635 nm ± 0.096 nm. These values indicate stable trajectories for both the models, with minimal positional deviations over time. This consistency confirms the conformational stability of the D117V model, suggesting a well-balanced system (Figure 5a).

Over a 100 ns MDs simulation, the native model exhibited an average RMSD of 0.670 nm ± 0.103 nm, whereas the D117V mutated model showed a comparable RMSD of 0.635 nm ± 0.096 nm. Considering a 95% confidence interval, these values suggest that the structural fluctuations of both the models remain within a similar range, indicating minimal impact of the D117V mutation on overall conformational stability.

#### Root-mean-square fluctuation

The residual flexibility of the PD-1 protein was analysed using RMSF, based on the positions of amino acid residues. The average RMSF values for the native and mutated PD-1 models were 0.309 nm ± 0.137 nm and 0.293 nm ± 0.129 nm, respectively, indicating similar overall flexibility between the two. However, higher fluctuations were observed in arginine residues at positions 30 and 161, as well as in the N-terminal region of the mutated protein compared to the native model (Figure 5b). Conversely, tryptophan at position 23 and arginine at position 231 showed greater stability in the mutated model. These variations suggest that the D117V polymorphism induces slight local fluctuations in residues distant from the mutation site without compromising overall structural stability.

### Radius of gyration

The protein compactness, shape and structural stability over time were evaluated by analysing the radius of gyration (*R*_g_) for both the native and D117V mutated models. After 40 ns of MD simulation, the mutated model exhibited a sudden increase in *R*_g_ fluctuations, in contrast to the native model. However, the average *R*_g_ values remained comparable between the two models, with the mutant showing an average of 2.296 nm ± 0.056 nm and the native model 2.194 nm ± 0.055 nm. These results indicate that despite localised fluctuations, the D117V mutation does not compromise the overall compactness and stability of the PD-1 protein structure throughout the MD simulation (Figure 5c).

### Solvent-accessible surface area

The surface area of a biomolecule interacting with solvent molecules was evaluated using the SASA parameter to determine its conformational stability in an aqueous medium. Our results show that the D117V polymorphism appears to abruptly fluctuate significantly between 30 ns and 50 ns. After 82 ns of simulation, both models (WT and D117V) stabilise at the same SASA value of 175 nm^2^. To summarise, native and mutated PD-1 protein structures maintained an almost similar average SASA value throughout the MD simulation with 180.997 (± 5.831) nm^2^ and 182.509 (± 7.001) nm^2^; respectively (Figure 5d). Given that the residues of the PD-1 protein are present on the surface of the protein, the presence of variants at this level induces a conservation of the accessibility of the surface of the solvent to the protein during the MD simulation.

### Hydrogen bonds

Hydrogen bonds (H-bonds) induce the formation of secondary and tertiary protein structures. Increases in the number of hydrogens induce stronger protein interactions. The MD simulation of the mutated model highlighted a notable increase in the hydrogen interaction with the native PD-1 protein, causing a stability of the bond between the two models over time, despite the conformational change. However, the average number of hydrogen bonds present during the MD simulation almost did not change and were 125.267 Å ± 8.672 Å for the native model and 127.685 Å ± 9.403 Å for the D117V mutated model in a 100 ns MD simulation (Figure 5e).

### Local structural changes

**FIGURE 6:**
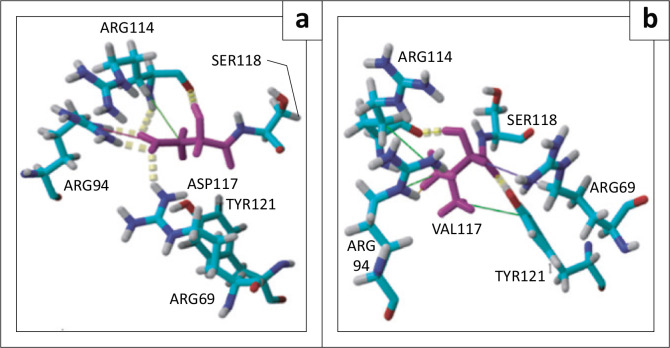
Structural model of the native (a) and mutated D117V (b) models after a 100 ns molecular dynamics simulation. Substituted residues are marked in magenta; residues involved in hydrophobic interactions are indicated in cyan. Hydrogen bonds are represented by yellow dotted lines, hydrophobic by green lines and ionic by blue lines.

A 100 ns MDs simulation, a comparison of the local structural changes induced by our D117V polymorphism was carried out with YASARA. Our results showed that there is a variation in the number of the different types of interactions between the native and mutated models. We found that in its native state, aspartic acid has two hydrogen bonds with the amino acids ARG94 and ARG114 and one hydrogen bond with ARG69. When valine replaced aspartic acid, one of the hydrogen bonds in ARG114 and the hydrogen bonds with ARG94 and ARG69 disappeared. We also observed a gain of hydrogen bonding with TYR121 (Figure 6b). Furthermore, in the native model, the ARG69 residue had an ionic bond with ASP117, whereas upon substitution of this aspartic acid with a valine this bond disappeared (Figure 6b). We also noticed that SER118 changed its conformation following this variation (Figure 6a). Moreover, the number of hydrophobic bonds has not really changed.

## Discussion

Bioinformatics tools are invaluable in analysing genetic information, providing insights into genome organisation, gene expression, sequence alignment, evolutionary relationships, MDs and the structure–function relationships of macromolecules.^32,33^ Several studies have leveraged these tools to advance scientific and medical research. For instance, in 2018, Kumar et al. used computational approaches to investigate the impact of missense mutations associated with ᴅ-2-hydroxyglutaric aciduria 2, elucidating molecular structural changes that can inform drug target development for this disease.^34^ Similarly, bioinformatics tools have been employed to study the association between Gaucher’s and Parkinson’s diseases, facilitating drug discovery for these conditions.^35^ Moreover, MD simulations have proven effective in evaluating the structural and functional properties of proteins and their assemblies.^36^

In this study, we conducted an *in silico* analysis of the human *PDCD1* gene to identify potentially deleterious nsSNPs and evaluate their impact on protein structure and stability. Our results showed that the two nsSNPs, D117V (rs77213099) and W286G (rs77510030), were identified by six out of seven predictive tools as highly deleterious, suggesting their potential to significantly affect PD-1 function.

Evolutionary conservation plays a critical role in determining the impact of mutations on human health.^37^ Using ConSurf, we analysed structural and functional residues by integrating evolutionary conservation data with solvent accessibility predictions. Our analysis revealed that nsSNPs in highly conserved regions are more likely to impair protein function compared to those in non-conserved regions^38,39^ underscoring their importance in maintaining PD-1’s structural and functional integrity.

Protein stability is essential for its proper function. Our findings suggest that the analysed variants, particularly D117V, may destabilise PD-1 at a functional level. The calculated *p*-values (Table 3) reinforced the reliability of these predictions, indicating that the D117V mutation could induce structural and functional alterations. However, it remains unclear whether these site-specific changes extend their impact beyond the protein level.

To explore these further, MD simulations were performed to compare the behaviour of the native and mutated forms of PD-1. However, PROCHECK validation of W286G model indicated that the mutated structure was positioned in an unauthorised or unfavourable region of the protein. This means that residue 286, both before and after mutagenesis, was in a stereochemically inappropriate location. This made the model structurally invalid. In contrast, the D117V mutant structure passed the POCHECK validation check, ensuring its stability and suitability for MD simulations. Therefore, the W286G model was deemed unsuitable and excluded from further analyses.

Analysis of RMSD, RMSF, *R*_g_, SASA and H-bond, following 100 ns MD simulation, showed that the D117V nsSNP does not influence the stability, flexibility and overall dimensions of the PD-1 protein. This allows us to conclude that this rs772130993 (D117V) polymorphism does not seem to influence the protein function.

The structure of the PD-1 protein was analysed to identify the different types of bonds between the different residues of the protein. These bonds play a major role in protein stability and folding. Each disruption of these interactions affects the structure and could interrupt the function of the PD-1 protein. The structure of this protein could also be affected by the differences in the physicochemical properties of amino acid variants caused by deleterious nsSNPs.^35,38,40^ Structural analysis of the D117V variant revealed changes, including the formation and/or loss of bonds, likely because of the distinct physicochemical properties of the substituted amino acids. Aspartic acid, an acidic residue, was replaced by valine, an aliphatic residue with different charges and hydrophobicity, potentially driving the observed structural and functional variations. This may be because of the fact that our polymorphism is located in a discrete region, in the middle of the component sequence of the extracellular region of our protein and therefore does not come into direct contact with the interactions of this protein. Also, the protection of this site is probably because of the fact that this amino acid is located in a helical region and therefore is stacked with adjacent residues, which gives it some resistance and protection. These results do not rule out the fact that this polymorphism, combined with other polymorphisms of the same or other regions, could disrupt protein function. Besides, predicting D117V as a deleterious SNP, but showing no significant functional change *via* MD is consistent with the literature. Some variants may appear harmful in predictions but demonstrate no substantial biological effects in dynamic environments.^41^ This reported lack of association between the rs772130993 (D117V) polymorphism and protein function would facilitate research into biomarker development and promise ease in the use of PD-1 inhibitors as treatment for HIV-infected subjects.

While multiple predictive tools identified D117V as a deleterious variant, MD simulations suggest that its structural and functional impact is minimal. This discrepancy highlights the limitations of static computational predictions, which primarily assess evolutionary conservation, physicochemical changes and generalised protein stability. In contrast, MD simulations provide a dynamic perspective, revealing that despite initial predictions, D117V does not significantly alter PD-1’s conformational stability or key functional interactions over time. Similar findings have been reported in other immune checkpoint proteins, where predicted deleterious mutations exhibited minimal phenotypic effects in dynamic cellular environments.^42^

While our findings suggest that the D117V variation has no significant functional effect, future research should test these predictions using such experimental approaches to clarify its biological impact. Furthermore, investigating the population-specific prevalence of these variations could provide insights into their role in disease susceptibility and immune modulation. Clinically, longitudinal studies assessing the correlation between PDCD1 polymorphisms and patient responses to PD-1 inhibitors in cancer or chronic infections could provide a foundation for personalised immunotherapy.^11^ Integrating these findings with proteomic and transcriptomic data may reveal broader biological implications of PD-1 polymorphisms, paving the way for optimised immunotherapeutic strategies.

To strengthen the clinical relevance of our findings, future studies should incorporate experimental validation of the predicted effects of PD-1 variants. Functional assays, such as Surface Plasmon Resonance or Isothermal Titration Calorimetry, could assess changes in ligand binding affinity,^43^ whereas structural analyses using X-ray Crystallography or cryo-Electron Microscopy would provide direct evidence of conformational alterations.^44^ Additionally, cellular assays evaluating immune activation and cytokine production in PD-1-expressing T cells could determine the physiological impact of these variants.^45^

## Conclusion

This *in silico* investigation aimed to study the deleterious nsSNPs of the PD-1 protein and examine their impact on the protein function. From 288 nsSNPs, two amino acid substitutions (D117V and W286G) were found to be deleterious, conserved and associated with decreasing stability and altering protein structure. More generally, the results of this prediction work will depend on the results, subsequently obtained on the impact of these polymorphisms on the protein function. Therefore, we realised a study of structural and functional modifications to directly determine the effect of these mutant proteins. Homology modelling and validation of our mutated structures only allowed us to retain the D117V variant. Our results demonstrated that this polymorphism does not have a strong impact on the structure of PD-1 and, therefore, on its function. This deduction could be useful for possible *in vitro* experimental studies and clinical trials in order to rule out the involvement of this polymorphism in the interactions of the PD-1 protein with its receptors. These findings suggest that anti-PD-1 therapy may offer a promising new approach for treating various mutation-associated diseases. While PD-1/PD-L1 inhibitors have shown considerable success in cancer treatment, their potential benefits for infectious diseases, such as HIV-1, hepatitis B or C virus patients, particularly, in improving T-cell function, are still being explored.^2,13^ However, deeper insights into PD-1 are needed to address potential treatment resistance.
