# Corrigendum: Functional and structural analysis of missense variants in the human PDCD1 Gene

**DOI:** 10.4102/jphia.v16i4.1574

**Published:** 2025-09-02

**Authors:** Hanâ Baba, Meryem Bouqdayr, Asmae Saih, Benson R. Kidenya, Mohamed A. Sesay, Simpson Addo, Lahcen Wakrim, Anass Kettani

**Affiliations:** 1 Biotechnology R&D Unit, Institut Pasteur du Maroc, Casablanca, Morocco; 2 Laboratory of Biology and Health, Faculty of Sciences Ben M’Sik, Hassan II University, Casablanca, Morocco; 3 Virology Unit, Immuno-virology Laboratory, Institut Pasteur du Maroc, Casablanca, Morocco; 4 Department of Biochemistry and Molecular Biology, Catholic University of Health and Allied Sciences, Mwanza, United Republic of Tanzania; 5 National Hepatitis Control Program, Ministry of Health, Freetown, Sierra Leone; 6 Government Hospital Viral Hemorrhagic Fever Research Site, Kenema, Sierra Leone; 7 Research, Statistics, and Information Management Directorate, Ministry of Health, Accra, Ghana

In the original publication, Baba H, Bouqdayr M, Abbad A, et al. Functional and structural analysis of missense variants in the human PDCD1 Gene. J Public Health Africa. 2025;16(4):a1348. https://doi.org/10.4102/jphia.v16i4.1348, the third author, Anass Abbad, has formally requested the removal of his name from the list of authors through a published correction, as he was not able to review or approve the final version of the manuscript prior to submission because of changes in institutional affiliation and limited communication at that time.

The authors apologise for this error. The correction does not change the study’s findings, its significance or overall interpretation of the study’s results or the scientific conclusions of the article in any way.

